# SP-DTI: subpocket-informed transformer for drug–target interaction prediction

**DOI:** 10.1093/bioinformatics/btaf011

**Published:** 2025-01-11

**Authors:** Sizhe Liu, Yuchen Liu, Haofeng Xu, Jun Xia, Stan Z Li

**Affiliations:** Thomas Lord Department of Computer Science, University of Southern California, Los Angeles, CA 90089, United States; Department of Quantitative and Computational Biology, University of Southern California, Los Angeles, CA 90089, United States; Thomas Lord Department of Computer Science, University of Southern California, Los Angeles, CA 90089, United States; School of Engineering, Westlake University, Hangzhou 310024, China; School of Engineering, Westlake University, Hangzhou 310024, China

## Abstract

**Motivation:**

Drug–target interaction (DTI) prediction is crucial for drug discovery, significantly reducing costs and time in experimental searches across vast drug compound spaces. While deep learning has advanced DTI prediction accuracy, challenges remain: (i) existing methods often lack generalizability, with performance dropping significantly on unseen proteins and cross-domain settings; and (ii) current molecular relational learning often overlooks subpocket-level interactions, which are vital for a detailed understanding of binding sites.

**Results:**

We introduce SP-DTI, a subpocket-informed transformer model designed to address these challenges through: (i) detailed subpocket analysis using the Cavity Identification and Analysis Routine for interaction modeling at both global and local levels, and (ii) integration of pre-trained language models into graph neural networks to encode drugs and proteins, enhancing generalizability to unlabeled data. Benchmark evaluations show that SP-DTI consistently outperforms state-of-the-art models, achieving an area under the receiver operating characteristic curve of 0.873 in unseen protein settings, an 11% improvement over the best baseline.

**Availability and implementation:**

The model scripts are available at https://github.com/Steven51516/SP-DTI.

## 1 Introduction

The process of drug discovery is extremely slow and costly, making the accurate prediction of drug–target interactions (DTI) crucial for the identification and development of new therapeutics. As the approval process for a drug by the Food and Drug Administration takes ∼12 years and costs over $1 billion (Van Norman 2016), there is a need for more efficient methods to screen and filter out compounds with low DTI, thereby reducing the sample size in subsequent phases of drug development. Traditional methodologies, such as molecular docking (Meng *et al.* 2011), which rely heavily on crystal structures and scoring functions (Pinzi and Rastelli 2019), are insufficient to address the increasingly intricate nature of emerging complex diseases. Additionally, machine learning models, such as SVM (Cortes and Vapnik 1995) and random forest (Breiman 2001), do not provide sufficiently high prediction accuracy, limiting their effectiveness in identifying potential DTIs (Voitsitskyi *et al.* 2023). Fortunately, the emergence of deep learning models has marked a shift in DTI prediction, moving beyond conventional approaches toward more efficient and integrative models.

Early methods obtained features solely from 1D sequence data and 2D molecular graphs, often using CNNs and GNNs. For example, DeepDTA (Öztürk *et al.* 2018) demonstrated the effectiveness of CNNs in extracting hidden representations from amino acid sequences and drug SMILES strings, and DeepConv-DTI (Lee *et al.* 2019) used convolution layers to capture the local residue patterns of protein subsequences. Other models have incorporated 2D molecular graphs of drug compounds. DEEPScreen (Rifaioglu *et al.* 2020) employed CNNs to learn the complex features of readily available 2D compound representations, and Tsubaki *et al.* (2019) used GNNs to handle drug molecular graphs predicted by RDkit (Landrum *et al.* 2021).

With the development of pre-trained language models and attention mechanism, DTI prediction models began to focus on more advanced techniques, such as substructure identification, protein and drug sequence pre-training, and addition of interaction layers (Lee and Nam 2022, Wei *et al.* 2022, Chatterjee *et al.* 2023). Notably, MolTrans (Huang *et al.*, 2021) employed the Frequent Consecutive Sub-sequence mining module to break the molecular sequences into sub-structures and included an interaction model that mimicked biological interactions; MocFormer (Zhang *et al.* 2023) used ESM-2 (Lin *et al.* 2022) and UNI-MOL (Lu *et al.* 2023, Zhou *et al.* 2023) to pre-train the protein and drug sequences, respectively, inputting them into a transformer with bilinear pooling; and DrugBAN (Bai *et al.* 2023) proposed a deep bilinear attention network with domain adaptation to learn the local interactions between drugs and proteins. Although these enhancements improved the DTI prediction performance, the models still relied only on 1D sequences and 2D molecular graphs. This approach provided limited information about the interactions, missing critical details such as the spatial arrangement of atoms and potential binding pockets on the proteins.

With the advancements in 3D structure prediction, the integration of spatial features has become crucial in DTI prediction. For instance, Ragoza *et al.* (2017) applied the CNN scoring function to evaluate DTI using protein–ligand complex datasets from pose prediction and virtual screening, thereby determining correct bindings. In addition to pre-determined 3D structures, recent models have utilized structure prediction tools such as RDKit (Landrum *et al.* 2021) and AlphaFold (Jumper *et al.* 2021) to convert drug and protein sequences into 3D structures. Drug3D-DTI (Liao *et al.* 2021) uses the RDKit package to generate 3D structures for small drug molecules but still uses 1D amino acid sequence for protein input. 3DProt-DTA (Voitsitskyi *et al.* 2023) incorporated AlphaFold to obtain a protein’s 3D structural data to enhance model adaptability, allowing it to be applied to proteins without crystal structures. The addition of spatial dimensionality to DTI models enables more detailed and precise interaction predictions. However, while integrating spatial features, these models are still primarily based on residue-level protein graphs and lack information regarding atomic-level binding pockets. To address this issue, AttentionSiteDTI (Yazdani-Jahromi *et al.* 2022) uses the convex hull algorithm (Saberi Fathi and Tuszynski 2014) to identify pockets and construct atomic-level pocket graphs, thereby improving the performance by providing more detailed structural information.

Despite extensive research efforts and several notable advances, the challenge of DTI prediction remains unresolved, as many studies report significant performance declines when tested on unseen protein splits and cross-domain datasets (Bai *et al.* 2023). These challenges persist primarily due to two key factors. First, the scope of labeled data is often limited, while vast amounts of unlabeled data remain underutilized. Although many studies have used pre-trained encoder models to generate latent-space molecular representations, these models often fail to incorporate graph-level knowledge for drugs and proteins. As a result, they overlook critical details related to stereochemistry and bonding, lacking the necessary physical and chemical knowledge to achieve a comprehensive molecular representation (Zhu *et al.* 2023). Second, the complexity of proteins, which can be represented at various levels such as sequences, amino acid graphs, and atom-level graphs, adds further challenges. Recent approaches have aimed to improve the quality of protein encoding by incorporating encoders for protein pockets, which are specific regions on the protein surface that serve as potential binding sites for drugs and can be modeled at the atom level (Wang *et al.* 2021a, Yazdani-Jahromi *et al.* 2022). However, these models often overlook the fact that pockets can be decomposed into subpockets, which more accurately represent how drugs bind at a finer level (Volkamer *et al.* 2010). Additionally, many analyses fail to assign importance scores to each pocket, missing valuable insights that could help the model better recognize the significance of different pockets.

In this study, we propose SP-DTI, which builds upon existing methodologies by introducing new modules for enhanced molecular representation, feature fusion, and interaction modeling.

- We introduce the Subpocket Modeling Module (SMM) to enable granular modeling of potential protein binding sites. We propose using the Cavity Identification and Analysis Routine (CAVIAR) (Marchand *et al.* 2021) to provide rank-based information for each pocket and to decompose each pocket into subpockets.
- We propose the Seq-Graph Fusion Module (SGFM) to integrate graph and sequence-level information. We applied pre-trained language models for both drugs and proteins, incorporating them as additional node features for both molecule graphs. To our knowledge, this is the first study to propose such a fusion method for both drugs and proteins in the DTI task. This approach effectively improves the generalizability of the model and enables a unified information representation for both drugs and proteins.
- We introduce a Subpocket-Informed Transformer, guided by the ranking of pockets, to integrate information at the subpocket, global protein, and global drug levels. This module effectively captures the interactions between molecules to improve binding prediction performance.

## 2 Materials and methods

We introduce SP-DTI, an end-to-end deep learning framework designed to address the challenges in DTI prediction outlined in the previous section. Before describing the framework in detail, we define the problem in Section 2.1, and justify the selection of base encoders in Section 2.2.

### 2.1 Problem definition

We frame the DTI prediction as a binary classification task. The objective was to determine the probability of interaction between the drug and target protein pairs. Drugs are represented by their SMILES notation D, which is a sequence of atomic and bond tokens derived from their molecular structure. Target proteins, denoted as A, are represented by amino acid token sequences. The task involves learning a function f(D,A)→{0,1} that maps each drug–target pair to a binary interaction score, where 0 represents no interaction and 1 represents an interaction.

### 2.2 Graph neural networks

Graphs provide a natural way to represent molecules, as they effectively capture key topological information such as bonds and neighborhood interactions (Tsubaki *et al.* 2019). As a result, graph-based methods are widely used in DTI tasks, often replacing sequence-based approaches like CNN-based or fingerprint-based methods (Shao *et al.* 2022). FlexMol further validates this approach empirically by evaluating various combinations of encoders and consistently demonstrating the superior performance of graph-based methods (Liu *et al.* 2024).

Prior studies have shown that among commonly used GNNs, including GAT, GCN, GIN, GINE, and GMF, no single model significantly outperforms others as a protein or ligand graph encoder in DTI tasks (Voitsitskyi *et al.* 2023). We extended the analysis to 3D ligand encoders, such as MGCN (Lu *et al.* 2019) and SchNet (Schütt *et al.* 2018), as detailed in Fig. S1, available as supplementary data at *Bioinformatics* online, and observed consistent results. Due to GCN’s simplicity and computational efficiency, we followed prior works in selecting it as the primary encoder (Wang *et al.* 2021b, Bai *et al.* 2023). Our encoder differs by integrating information from a pre-trained language model, which is discussed in detail in Section 2.3.2.

### 2.3 Model

**Figure 1.**
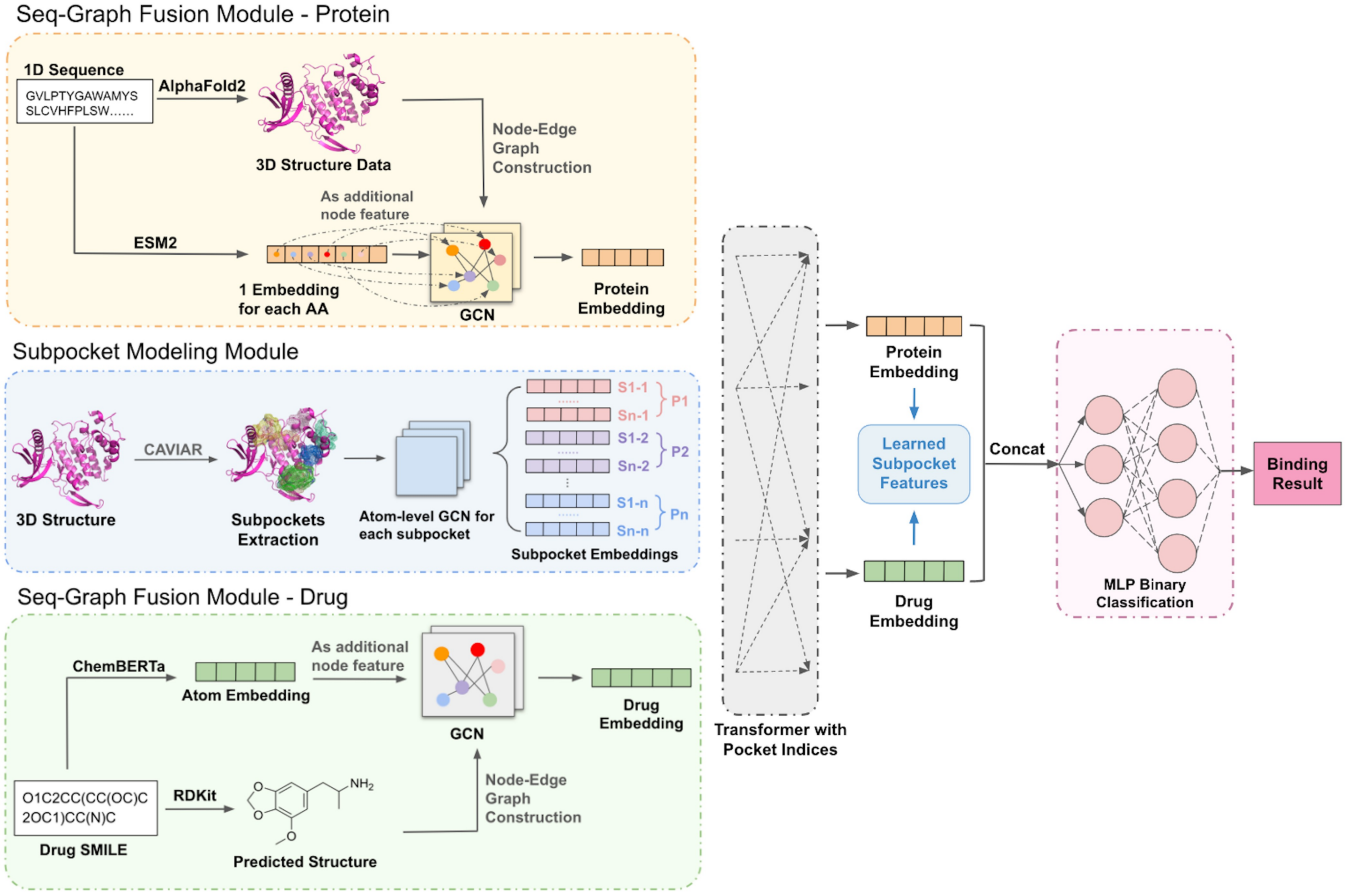
Overview of SP-DTI. Our proposed framework includes the following main steps: (i) Preprocessing, which involves generating 3D protein structures using AlphaFold and identifying subpockets of proteins using CAVIAR. Please refer to Fig. 2 for subpockets identification illustration and the original CAVIAR paper for detailed algorithm. (ii) Seq-Graph Fusion, where ESM-2 and ChemBERTa are used to create embeddings for proteins and drugs, respectively, and these embeddings are added as additional node features for GCNs. (iii) Subpocket Encoding, which constructs atom-level graphs for each subpocket and processes them through a shared weight GCN. (iv) Interaction Modeling, using a transformer to model the interactions between subpockets, global protein representations, and global drug representations. (v) Prediction, which concatenates the drug and protein representations from the transformer to predict the interaction likelihood for the drug–protein pair.

Our SP-DTI model consists of three parts: SMM, SGFM, and Interaction Module. An overview of the proposed model is shown in Fig. 1.

#### 2.3.1 Subpocket modeling module

The SMM aims to capture the intricate interactions between drugs and proteins at the atomic level. This is achieved by identifying and modeling subpockets, which are smaller regions within larger protein binding pockets that drug molecules can potentially attach to. Volkamer *et al.* (2010) suggested that subpockets provide a more accurate representation of real ligand-binding regions because ligands are often predominantly contained within a single subpocket, thereby achieving higher pocket coverage.

**Figure 2.**
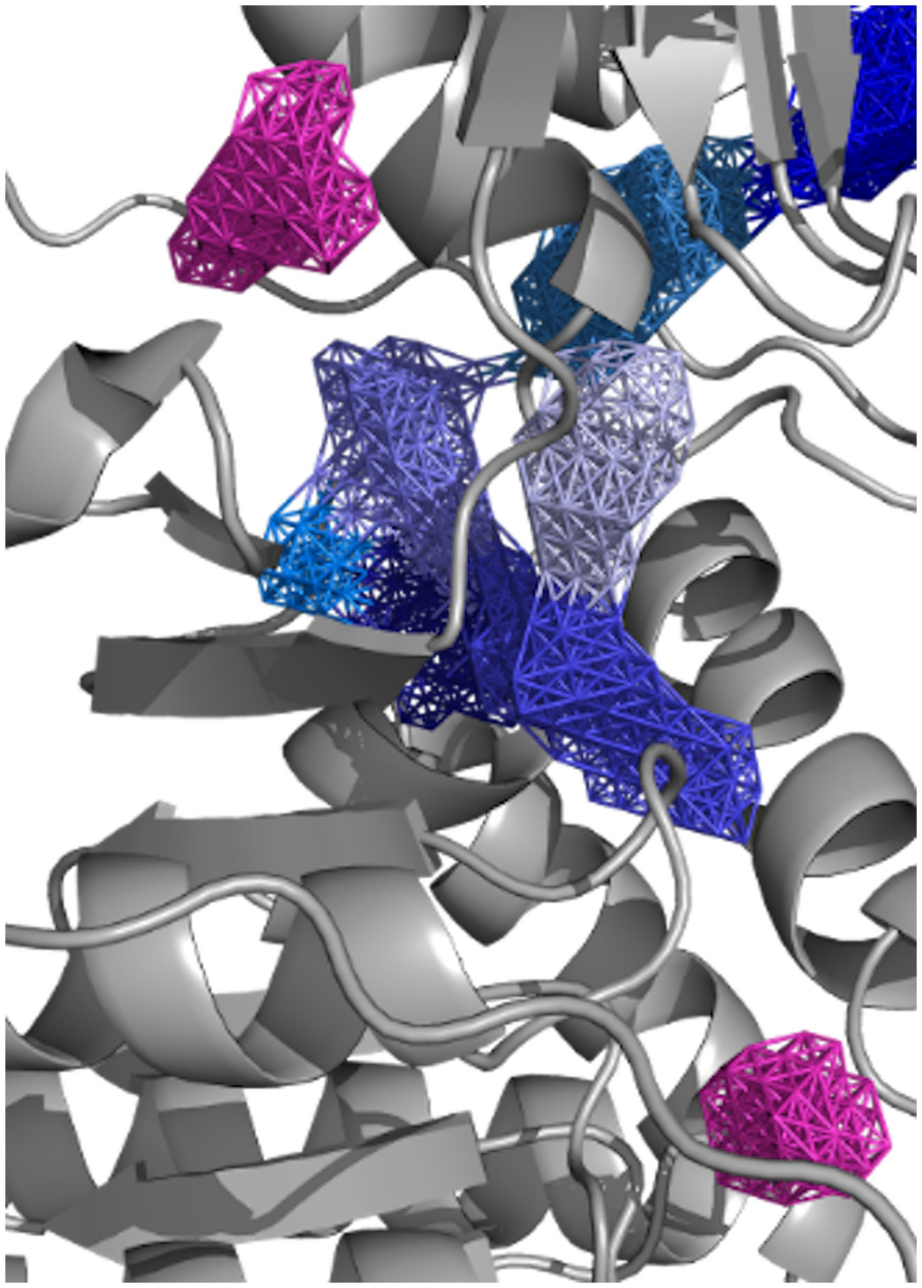
Illustration of subpockets identified by the CAVIAR algorithm. The central pocket is divided into multiple subpockets. A higher CAVIAR score indicates a greater likelihood that the subpocket will become a ligand binding site. The pockets on the top left and bottom right cannot be decomposed into smaller subpockets and therefore require the entire pocket as the input.

The three-dimensional structures of the proteins were obtained in the Protein Data Bank (PDB) format using AlphaFold2 (Jumper *et al.* 2021). Subsequently, we employed the CAVIAR (Marchand *et al.* 2021) algorithm to identify potential binding pockets and further dissect them into subpockets. For each identified pocket $p_{i}$, CAVIAR assigns a score $c_{i}$ that quantifies the likelihood that the pocket is a viable binding site for ligands. Figure 2 presents an example of the subpockets identified using CAVIAR.

We defined $P=\{p_{1},p_{2},\ldots ,p_{n}\}$ as the set of all identified pockets within a protein structure, where pockets are indexed such that a lower index corresponds to a higher CAVIAR score, i.e. $c_{i}\geq c_{j}$ for i<j.

For each pocket $p_{i}$, let $S_{i}$ denote its set of subpockets. We introduce a constant M∈N, representing the maximum allowable total number of subpockets used as the model input. The collective set $S=\overset{n}{\underset{i=1}{\cup }}S_{i}$ aggregates subpockets from all pockets, subject to the constraint |S|≤M. Each subpocket $s_{i}$ is assigned a rank $k_{i}$, such that $s_{i}\in S_{k_{i}}$.

An individual graph was generated for every subpocket within set S. Unlike the cohesive structure of an entire protein, the subpockets may be composed of several disconnected segments. To ensure clarity of representation, smaller fragments containing fewer than five atoms were omitted, retaining only the atoms in the principal fragments. If |S|<M, placeholder graphs comprising a single node with null embedding are added to maintain the consistency.

Finally, each of the M graphs was processed using a GCN with max and weighted pooling. The same set of weights was applied to all the graphs to produce an M×d embedding. This embedding represents a detailed summary of the features of the subpockets.

#### 2.3.2 Seq-Graph fusion module

The SGFM was designed to enhance the GNN’s encoding abilities by leveraging large language models. We represent the structure of a protein as a residue-level graph, denoted by G=(E,V), where V and E represent nodes and edges, respectively. V corresponds to amino acid residues, and E is established based on three types of interactions: peptide bonds, hydrogen bonds, and the K-nearest neighbors algorithm (with k=5).

For the node features of the protein graph, we feed amino acid sequences into the protein language model ESM-2 (Lin *et al.* 2023), a state-of-the-art model trained on ∼65 million unique sequences. This process generated features for each residue, represented as $h\in R^{N\times 1280}$. To enrich these features, we concatenated them with additional biological information about amino acids, specifically their electrostatic properties, which serve as node attributes for the protein graph.

Expanding this approach to drug molecules, we first constructed a drug graph from the SMILES representation using RDKit (Landrum *et al.* 2021). The SMILES strings were then processed using ChemBERTa (Ahmad *et al.* 2022), a specialized language model trained on 77 million SMILES strings. This yields features $h^{\prime }\in R^{N\times 384}$ for each SMILES token, from which we extract only the features corresponding to the actual atoms to be used as node attributes for the drug graph. Similar to the protein graph, we augmented these features with the chemical properties of the atoms for a more comprehensive representation.

Finally, the constructed protein and drug graphs were processed using distinct GCNs with max and weighted pooling layers. This setup yielded a unified representation with dimensions d for proteins and drugs.

#### 2.3.3 Interaction module

The Transformer Interaction Module models drug–protein interactions by incorporating both overall structural and subpocket-specific details. To begin, it combines drug, protein, and subpocket embeddings into a matrix $X\in R^{(M+2)\times d}$, where M denotes the number of subpockets, and d is the embedding dimension.

To capture positional relationships within this matrix, we modified the standard transformer encoder to include a positional encoding scheme based on pocket indices, establishing links between each subpocket and its respective pocket, while also conveying the relative importance of each pocket. The positional encoding function is defined as follows:


$$\text{Pos}(x)=\{\begin{array}{cc} k_{i}, & \text{if}\;x=s_{i}\;(\text{subpocket})\\ 0, & \text{if}\;x=\text{drug}\;\text{or}\;\text{protein} \end{array}$$

In this formulation, $k_{i}$ represents a unique positional value assigned to each subpocket $s_{i}$, capturing both the pocket association and the subpocket’s rank concerning drug-binding potential. Drug and protein embeddings are assigned a positional encoding of zero, reflecting their distinct, non-sequential roles in the interaction module. The encoder then performs multi-head attention on X, producing an updated matrix $X^{\prime }\in R^{(M+2)\times d}$. This updated representation is pivotal for capturing the interactions among the drug, protein, and subpocket embeddings.

To determine the probability of interaction, the drug and protein embeddings are concatenated to form a single embedding $O\in R^{2d}$, which is enriched with knowledge of the pocket information captured through the attention mechanism. This consolidated embedding vector is then passed through a multilayer perceptron (MLP). A linear layer, parameterized by a weight matrix $W_{o}$ and bias vector $b_{o}$, processes the output of the MLP: $\sigma (o)=\frac{1}{1+\text{exp}(-o)}$, where $o=W_{o}\cdot \text{MLP}(O)+b_{o}$. This probability score indicates the potential for interaction between the drug and the target protein.

During training, the network is optimized using the binary cross-entropy loss, defined as L=−[Y log(P)+(1−Y) log(1−P)], where Y is the ground truth label and P represents the predicted probability of interaction (Huang *et al.* 2021). All parameters are updated jointly via backpropagation.

## 3 Experiments and results

### 3.1 Implementation

SP-DTI was implemented using PyTorch (Paszke *et al.* 2019) and FlexMol (Liu *et al.* 2024), a toolkit for efficiently constructing and evaluating DTI models. The models were trained with a batch size of 32 for 30 epochs using the Adam optimizer with a learning rate of 0.0001. The model typically converges between 12 and 15 epochs. All the experiments were conducted using an NVIDIA V100 GPU. For the SMM, the maximum number of supported subpockets was set to 30. In the transformer interaction layer, the input embedding size was 128, with four attention heads and an intermediate dimension of 512. The dropout rate was set at 0.1. The maximum number of subpockets, M, was set to 30. A full list of hyperparameters is provided in Table S1, available as supplementary data at *Bioinformatics* online.

### 3.2 Experimental setup

#### 3.2.1 Dataset

We utilized the same datasets and preprocessing methods as the MolTrans framework to evaluate the DTI performance (Huang *et al.* 2021). Our setup also integrated AlphaFold2-generated structures to enrich the datasets.

Specifically, BIOSNAP includes 4 510 drugs and 2 181 protein targets, resulting in 13 741 DTI pairs from DrugBank (Marinka Zitnik *et al.* 2018). Notably, BIOSNAP includes only positive DTI pairs, while negative pairs are generated by sampling from unobserved interactions. The DAVIS dataset contains Kd values for 68 drugs and 379 proteins (Davis *et al.* 2011), with pairs below a Kd of 30 units classified as positive. An equal number of negative pairs were added for balanced training. The dataset statistics after pre-processing are presented in Table 1.

**Table 1.** Description and statistics of the processed benchmark datasets.

Dataset	# Drugs	# Proteins	# Positive interactions	# Negative interactions
BIOSNAP	4510	2181	13 741	13 741
DAVIS	68	379	1506	9597

#### 3.2.2 Metrics

We used area under the receiver operating characteristic curve (ROC-AUC) and area under the precision–recall curve (PR-AUC) to measure binary classification performance. Additionally, we evaluated sensitivity and specificity, using the threshold that achieved the best F1 score on the validation set.

#### 3.2.3 Evaluation strategies

The dataset was divided into training, validation, and testing sets. To thoroughly assess the robustness of the model, we employed three splitting strategies: random split, unseen drug/protein split, and cross-domain split. The specific methods used for each split are detailed in the corresponding sections. The best-performing model was selected based on the ROC-AUC performance on the validation set.

### 3.3 Baseline models

We evaluated our model against several state-of-the-art models in DTI prediction, selected for their prominence in the field and their diverse methodological approaches:

1. **Traditional ML methods**: SVM (Cortes and Vapnik 1995), RF (Breiman 2001), and LR (Cox 1958) were applied to the concatenated fingerprint ECFP4 and Protein Sequence Composition (PSC) (Cao *et al.* 2013) features.
2. **GNN-CPI** (Tsubaki *et al.* 2019): A graph neural network was employed to encode drugs, and a CNN was used to encode proteins. The latent vectors were concatenated for interaction prediction.
3. **DeepDTA** (Öztürk *et al.* 2018): CNNs were used to process both SMILES strings and protein sequences, extracting local residue patterns.
4. **DeepConv-DTI** (Lee *et al.* 2019): CNNs and a global max pooling layer were utilized to capture local patterns of varying lengths in protein sequences, and a fully connected layer was used to process the drug fingerprint ECFP4.
5. **MolTrans** (Huang *et al.* 2021): Sub-structural pattern mining and an augmented transformer encoder were employed to model the semantic relations among sub-structures.
6. **DrugBAN** (Bai *et al.* 2023): An interpretable bilinear attention network was applied to model local interactions between drug molecular graphs and target protein sequences.
7. **3DProtDTA** (Voitsitskyi *et al.* 2023): AlphaFold’s structure predictions and graph representations of proteins were utilized for drug–target affinity prediction, with graph neural networks used to process these representations.

### 3.4 Testing on random split

**Figure 3.**
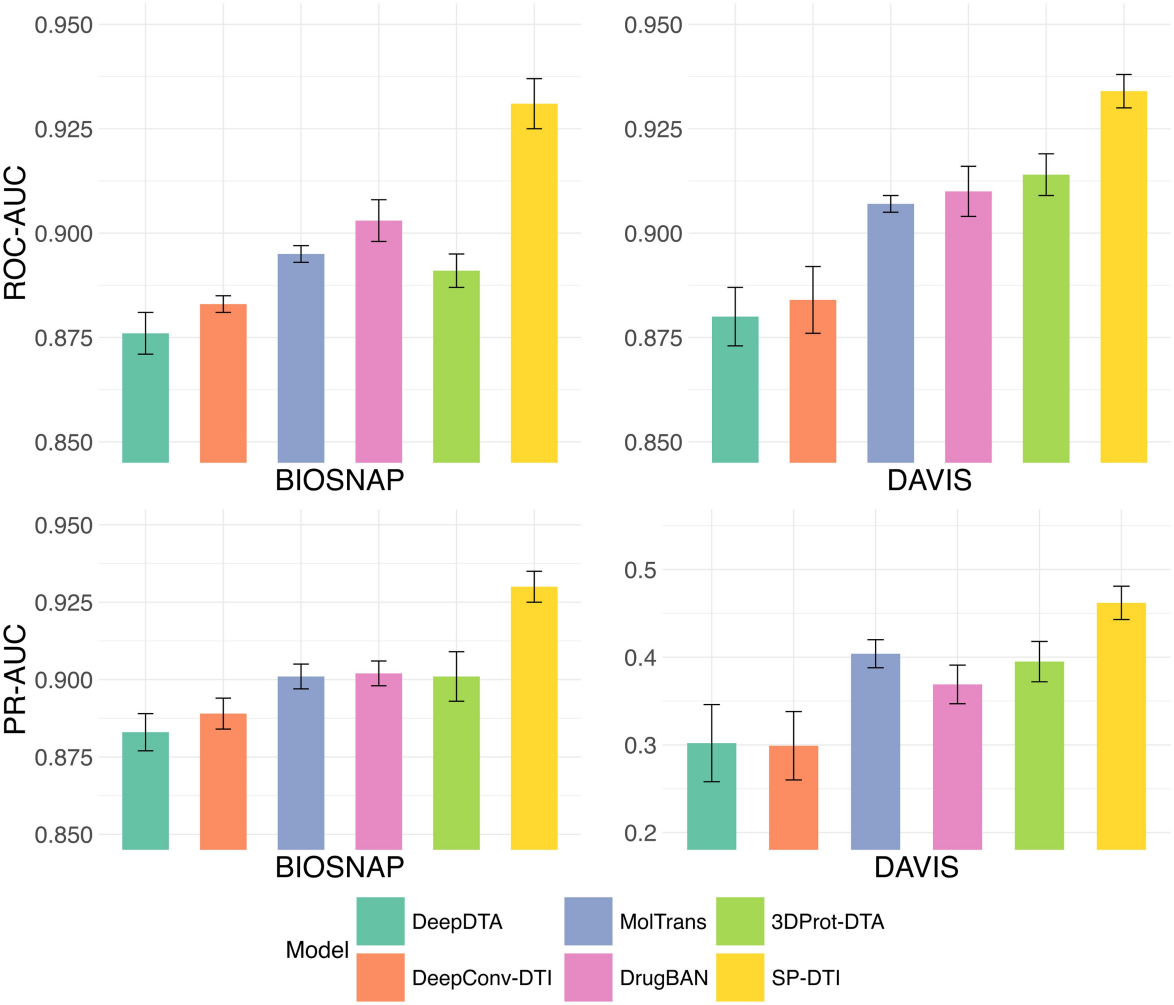
Comparison of SP-DTI with the top five baseline models: (Top) Area Under the Curve (AUC) for random splits in the test set; (Bottom) Precision-Recall AUC (PR-AUC) for random splits in the test set.

For both the DAVIS and BIOSNAP datasets, we conducted a random split in the ratio of 7:2:1 for training, validation, and testing. The experimental results are presented in Table 2. Figure 3 shows a comparison of SP-DTI with the top five baselines. As shown, SP-DTI consistently outperforms all baselines in terms of ROC-AUC and PR-AUC across both datasets. Notably, SP-DTI demonstrated a relative percentage improvement of up to 14% in the PR-AUC compared to the best-performing baseline on the DAVIS dataset.

**Table 2.** Performance comparison on BIOSNAP and DAVIS random split.

Method	ROC-AUC	PR-AUC	Sensitivity	Specificity
Dataset 1: BIOSNAP				
LR	0.846±0.004	0.850±0.011	0.755±0.039	0.800±0.018
SVM	0.862±0.007	0.864±0.004	0.777±0.011	0.711±0.042
RF	0.860±0.005	0.886±0.005	0.804±0.005	0.823±0.032
GNN-CPI	0.879±0.007	0.890±0.004	0.780±0.014	0.819±0.012
DeepDTA	0.876±0.005	0.883±0.006	0.781±0.015	0.824±0.012
DeepConv-DTI	0.883±0.002	0.889±0.005	0.770±0.023	0.832±0.016
MolTrans	0.895±0.002	0.901±0.004	0.775±0.032	0.851 ± 0.014
DrugBAN	0.903 ± 0.005	0.902 ± 0.004	0.820±0.021	0.847±0.010
3DProt-DTA	0.891±0.004	0.901±0.008	0.826 ± 0.017	0.806±0.021
SP-DTI	**0.931** ± **0.006**	**0.930** ± **0.005**	**0.863** ± **0.024**	**0.857** ± **0.011**
Dataset 2: DAVIS				
LR	0.835±0.010	0.232±0.023	0.699±0.051	0.842±0.033
SVM	0.838±0.006	0.256±0.017	0.716±0.041	0.837±0.018
RF	0.845±0.008	0.253±0.020	0.735±0.038	0.859±0.021
GNN-CPI	0.840±0.012	0.269±0.020	0.696±0.047	0.842±0.039
DeepDTA	0.880±0.007	0.302±0.044	0.764±0.045	0.865±0.020
DeepConv-DTI	0.884±0.008	0.299±0.039	0.754±0.040	0.880±0.024
MolTrans	0.907±0.002	0.404 ± 0.016	0.800 ± 0.022	0.876±0.013
DrugBAN	0.910±0.006	0.396±0.022	0.794±0.041	0.885±0.023
3DProt-DTA	0.914 ± 0.005	0.395±0.023	0.799±0.041	**0.901** ± **0.018**
SP-DTI	**0.934** ± **0.004**	**0.462** ± **0.019**	**0.837** ± **0.036**	0.884 ± 0.015

Bold indicates the best model, and underline indicates the second-best model.

### 3.5 Testing on unseen drug/protein split

**Figure 4.**
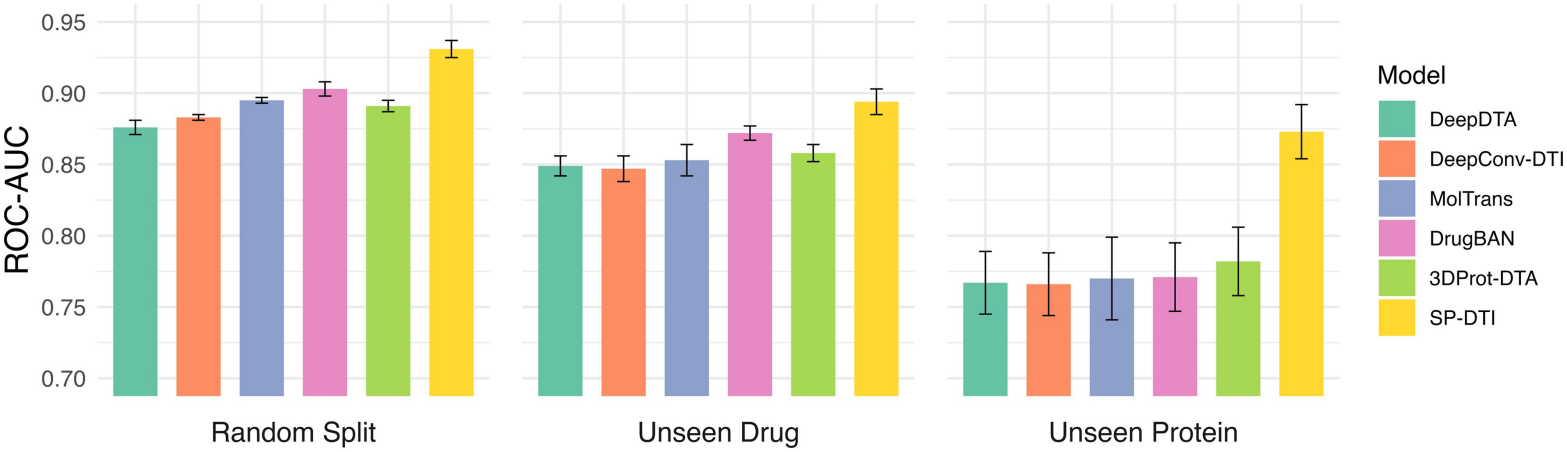
Comparison of SP-DTI with the top five baseline models across different settings (random splits, unseen drug, and unseen protein). There is a drop in performance for each model when encountering unseen drugs or proteins, but SP-DTI has the highest ROC-AUC under all settings and experiences the lowest drop.

Unseen drug and target settings are crucial for assessing the predictive power of the model in real-world scenarios where novel drug–target pairs are constantly emerging. The split method was adapted from MolTrans. Specifically, 20% of the drug/target proteins and all DTI pairs associated with these drugs and targets were selected as the test set. Table 3 and Figure 4 show that SP-DTI has a competitive performance against SOTA deep learning baselines in both settings. We observed that all other baselines experienced a significant drop in relative performance for unseen proteins (>12%), whereas our method experienced only a 6% drop. One reason SP-DTI performs well in the unseen protein setting is the integration of pre-trained ESM features. These features capture comprehensive evolutionary and structural information from large-scale protein datasets, allowing the model to generalize effectively to unseen proteins.

**Table 3.** Performance on BIOSNAP unseen drug/protein split.

Settings	DeepDTA	DeepConv-DTI	MolTrans	DrugBAN	3DProt-DTA	SP-DTI
Unseen Drugs	0.849 ± 0.007	0.847 ± 0.009	0.853 ± 0.011	0.872 ± 0.005	0.858 ± 0.006	**0.894** ± **0.009**
Unseen Protein	0.767 ± 0.022	0.766 ± 0.022	0.770 ± 0.029	0.771 ± 0.024	0.782 ± 0.024	**0.873** ± **0.019**

Bold indicates the best model, and underline indicates the second-best model.

### 3.6 Testing on BIOSNAP cross-domain split

Cross-domain testing, in which the test set is both unseen and outside the learned distribution, presents the most challenging scenario. We adopted the cross-domain setup from the DrugBAN paper (Bai *et al.* 2023), utilizing single-linkage clustering for drugs and proteins based on ECFP4 fingerprints and pseudo amino acid composition (PSC) (Cao *et al.* 2013). After clustering, we randomly select 60% of the drug clusters and 60% of the protein clusters. All drug–target pairs between selected drugs and proteins were considered source domain data, while pairs involving the remaining clusters formed the target domain data. The training set comprised all the labeled data from the source domain, and the test set consisted of 20% of the target domain data. Table 4 demonstrates the strength of SP-DTI in generalizing the prediction performance across domains.

**Table 4.** Comparison of the cross-domain performance on BIOSNAP.

	**MolTrans_cdan_** ^a^	**DrugBAN_cdan_** ^a^	3DProt-DTA	SP-DTI
**ROC-AUC**	0.656 ± 0.028	0.684 ± 0.026	0.663 ± 0.031	**0.773** ± **0.025**

a _cdan_ indicates training using Conditional Domain Adversarial Network (CDAN) with additional unlabeled data from target domain data. Bold indicates the best model, and underline indicates the second-best model.

### 3.7 Model interpretation

**Figure 5.**
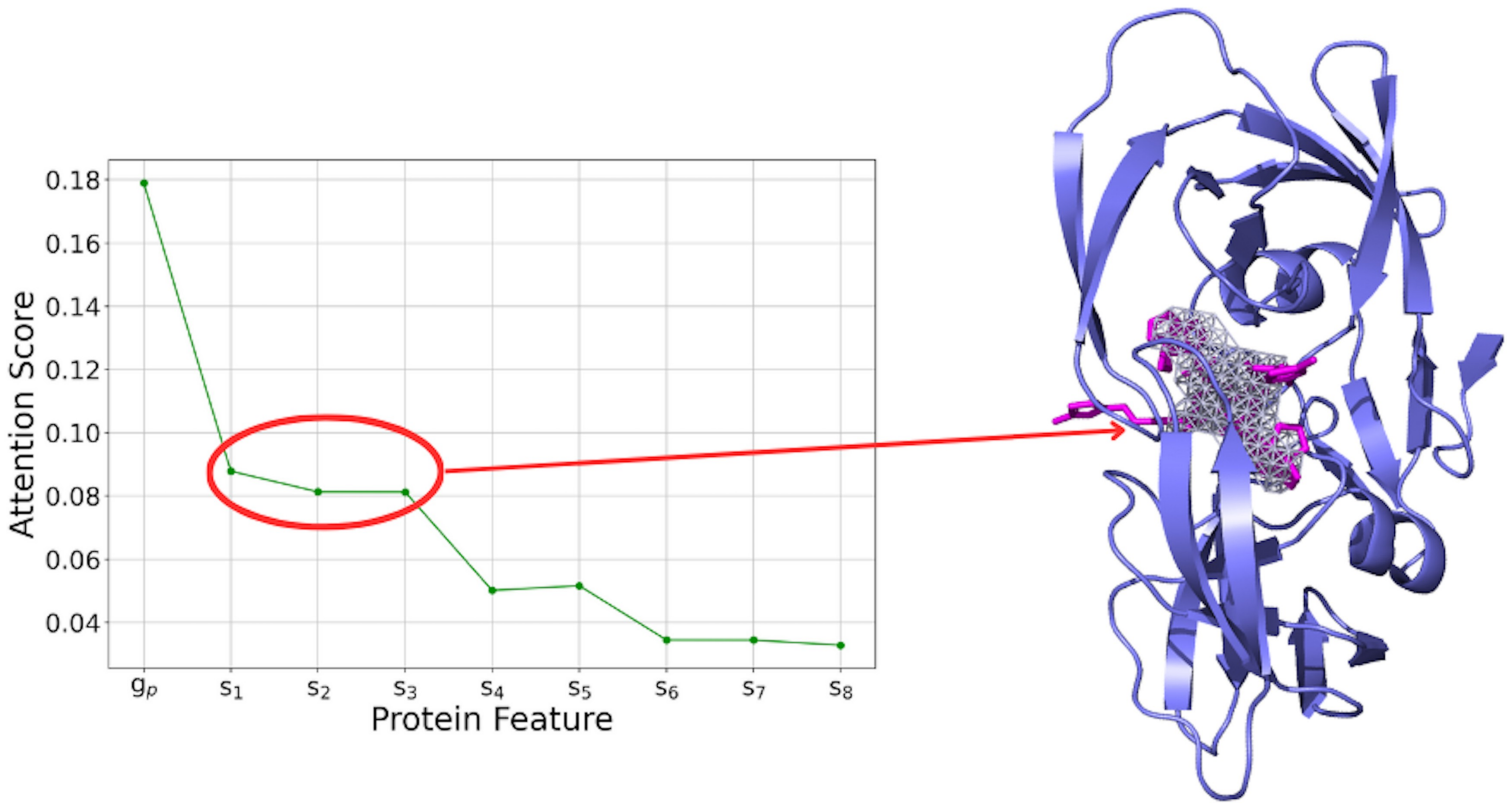
(Left) A line plot of self-attention mechanism weights for each protein feature in the proposed method using HIV protease D545701 as the protein and GW0385 as the ligand. Here, $g_{p}$ denotes the global protein feature, and $s_{i}$ represents the *i*-th subpocket. (Right) Projected regions representing the top three subpockets with the highest attention weights, which correspond precisely to the actual binding positions of the ligand.

In this work, the attention mechanism allows the model to predict which protein binding sites are most likely to bind to a given ligand. These probabilities were represented by the attention matrix generated by the model. For our case study, we selected the crystal structure of HIV protease D545701 bound to GW0385 (PDB: 2FDD). Using CAVIAR, we identified three subpockets within this structure. Additionally, we included five randomly selected regions from the unbound parts of the protein to simulate potential false positives identified by CAVIAR. The attention visualization is presented in Fig. 5, demonstrating that during prediction, global protein embedding receives more attention than all subpockets; notably, the three subpockets with the highest attention weights correspond precisely to the experimentally verified binding sites. We further provide a heatmap representing the attention matrix in Fig. S2, available as supplementary data at *Bioinformatics* online. This demonstrates that the interaction module not only improves model performance but also enhances model interpretability.

### 3.8 Ablation study

This ablation study aimed to determine the contribution of each component to our model. We assessed their impact on the overall performance by systematically removing or altering specific layers or features. The configurations tested were as follows:

- **W/o subpocket:** Removing the subpocket encoder.
- **Pocket:** Using pockets generated by the convex hull algorithm instead of subpockets as the input.
- **W/o pre-train:** Excluding the additional node features from pre-trained language models.
- **W/o interaction:** Removing the transformer module used to model the interaction and directly concatenating features from the three encoders.
- **W/o fusion:** Concatenation is used to integrate features from large language models and graph features instead of SGFM.

From Table 5, it is evident that features from the pre-trained language models have the strongest influence on performance. The subpocket encoder, interaction layer, and fusion module also significantly contribute to the overall performance of the model. Specifically, removing the subpocket module or replacing it with a pocket approach both result in a decrease in performance.

**Table 5.** Results of the ablation study on BIOSNAP.

Settings	ROC-AUC	PR-AUC
SP-DTI	0.931 ± 0.006	0.930 ± 0.005
w/o subpocket	0.923 ± 0.005	0.924 ± 0.003
pocket	0.926 ± 0.004	0.923 ± 0.007
w/o pre-train	0.913 ± 0.003	0.911 ± 0.004
w/o interaction	0.920 ± 0.005	0.921 ± 0.002
w/o fusion	0.925 ± 0.006	0.926 ± 0.006

### 3.9 Discussion

Our results show that SP-DTI outperforms baseline models, particularly in unseen protein and cross-domain settings. Baseline models struggle in these scenarios due to overfitting in protein encoding. Unlike drugs, which typically consist of fewer than a hundred atoms, proteins can contain tens of thousands. This complexity, combined with the limited number of unique proteins in DTI datasets (e.g. 379 in DAVIS and 2181 in BIOSNAP), makes it challenging for deep learning models to achieve generalizable representations. This explains why baseline models exhibit only minor performance drops in unseen drug settings but experience more significant declines in unseen protein or cross-domain settings.

To address this, our encoder leverages pretrained language models trained on millions of unlabeled protein sequences and integrates them with graph neural networks to enhance the generalizability of protein encodings while preserving important geometric information. Additionally, our approach incorporates subpocket information, enabling detailed atom-level encoding of proteins alongside global amino-acid level graphs. This dual-level encoding captures both the broader structural context and the fine-grained details of protein interaction sites, resulting in a more accurate and biologically relevant representation. The contribution of each component to our model is validated through ablation studies.

The key contributions of SP-DTI include its high prediction accuracy, model robustness, and interpretability of results. In real-world applications, where the chemical and genomic spaces are vast, DTI pairs are often dissimilar to the training set (Bai *et al.* 2023). SP-DTI demonstrates not only strong performance in random split settings but also robustness in unseen and cross-domain settings, highlighting its potential for real-world applicability. By using deep learning to identify DTI pairs, SP-DTI can significantly narrow the search space for compound candidates, thereby reducing the costs associated with pharmaceutical research.

A further strength of SP-DTI is to enable interpretation, which is crucial for drug discovery. Through the use of attention maps from the transformer module, SP-DTI provides insights into the specific protein binding subpockets most likely to interact with a given ligand, as demonstrated in our case study, allowing scientists to understand why a particular interaction is predicted. This transparency is believed to reduce the risk of false positives and accelerate the drug discovery process.

## 4 Conclusion

In this study, we introduce SP-DTI, a subpocket-informed transformer model designed for DTI prediction. The incorporation of subpocket information and the SGFM effectively enhanced the predictive power of the model. Our comprehensive evaluations in in-domain and cross-domain settings demonstrate that SP-DTI consistently outperforms state-of-the-art baselines in all cases, providing improved accuracy and robustness.

### Code and data availability

SP-DTI is open-sourced and available on GitHub at https://github.com/Steven51516/SP-DTI. The random and unseen drug/protein data splits for the DAVIS and BioSNAP datasets were obtained from the MolTrans repository at https://github.com/kexinhuang12345/MolTrans. The cross-domain split for the BioSNAP dataset were obtained from the DrugBAN repository at https://github.com/peizhenbai/DrugBAN.

## Supplementary Material

btaf011_Supplementary_Data
