# Supplementary material for: SP-DTI: subpocket-informed transformer for drug–target interaction prediction
Source: Bioinformatics. 2025 Jan 11;41(3):btaf011. doi: 10.1093/bioinformatics/btaf011 (PMC11886779; doi:10.1093/bioinformatics/btaf011)
Supplement: btaf011_Supplementary_Data [file btaf011_supplementary_data.zip › 82861_supp-spdti.pdf]

Supplementary Materials to SP-DTI: Subpocket-Informed Transformer for Drug-Target Interaction Prediction

1. OPIMIZED HYPERPARAMETER

Hyperparameter	Value
Batch Size	32
Learning Rate	0.0001
Number of Epochs	25
Early Stopping Patience	7
GCN Hidden Layer Dimensions	[64, 64, 64]
GCN Pooling Strategy	max_and_mean
Maximum Number of Subpockets	30
Transformer Dropout Rate	0.1
Transformer Embedding Dimension	128
Transformer Intermediate Dimension	512
Transformer Number of Attention Heads	4
Classification Head Layer Dimensions	[256, 128, 64]

Table S1. Optimized hyperparameters for SP-DTI.

2. TRAINING PARAMETERS AND INFERENCE TIME

Model	Inference Time (s)	Trainable Parameters
SP-DTI	989.99	1,073,851
w/o Transformer	892.29	705,467
w/o Transformer & Subpocket Module	805.06	612,985

Table S2. Inference time and trainable parameters for different models. *w/o Transformer* refers to the model configuration where outputs from all three encoders are concatenated, with mean pooling applied to the subpocket encoder. *w/o Transformer & Subpocket Module* refers to the model configuration where the outputs from the drug and protein encoders are concatenated, excluding the subpocket module. The inference time is calculated using the test set of the BIOS-NAP dataset on a single V100 GPU.

3. JUSTIFICATION OF GNN CHOICE

Prior studies have shown that for drug-target interaction tasks, no single GNN model consistently outperforms others as a protein graph encoder among popular options like GAT, GCN, GIN, GINE, and GMF [1]. Similarly, for ligand graph processing, there is no definitive best choice among GNN variants. To further investigate this, we extended the comparison to more advanced ligand encoders, such as MGCN [2] and SchNet [3]. Specifically, we fixed the protein encoder to a simple GCN and evaluated the performance of different ligand encoders. The outputs from the two encoders were concatenated and fed into an MLP for prediction. Our results, presented in Fig. S1, are consistent with previous findings: none of the tested GNNs, including MGCN and SchNet, significantly outperformed the simpler GCN.

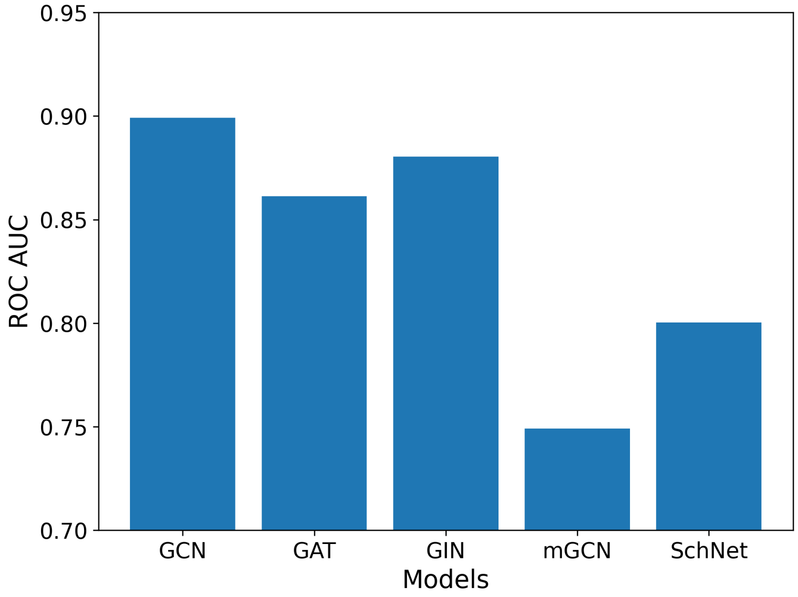

Fig. S1. ROC-AUC values for five ligand encoders (GCN, GAT, GIN, MGCN, and SchNet) on the Davis dataset for the drug-target interaction task. The experiment used a fixed GCN as the protein encoder.

4. ADDITIONAL PERFORMANCE METRICS

Table S3. Full Performance table of SP-DTI in Unseen Drug, Unseen Protein, and Cross Domain Settings

Method	ROC-AUC	PR-AUC	Sensitivity	Specificity
Unseen-drug	0.894 ± 0.009	0.909 ± 0.012	0.825 ± 0.027	0.804 ± 0.022
Unseen-prot	0.873 ± 0.019	0.869 ± 0.019	0.869 ± 0.032	0.707 ± 0.019
Cross Domain	0.773 ± 0.025	0.776 ± 0.018	0.611 ± 0.041	0.841 ± 0.039

5. ATTENTION HEATMAP

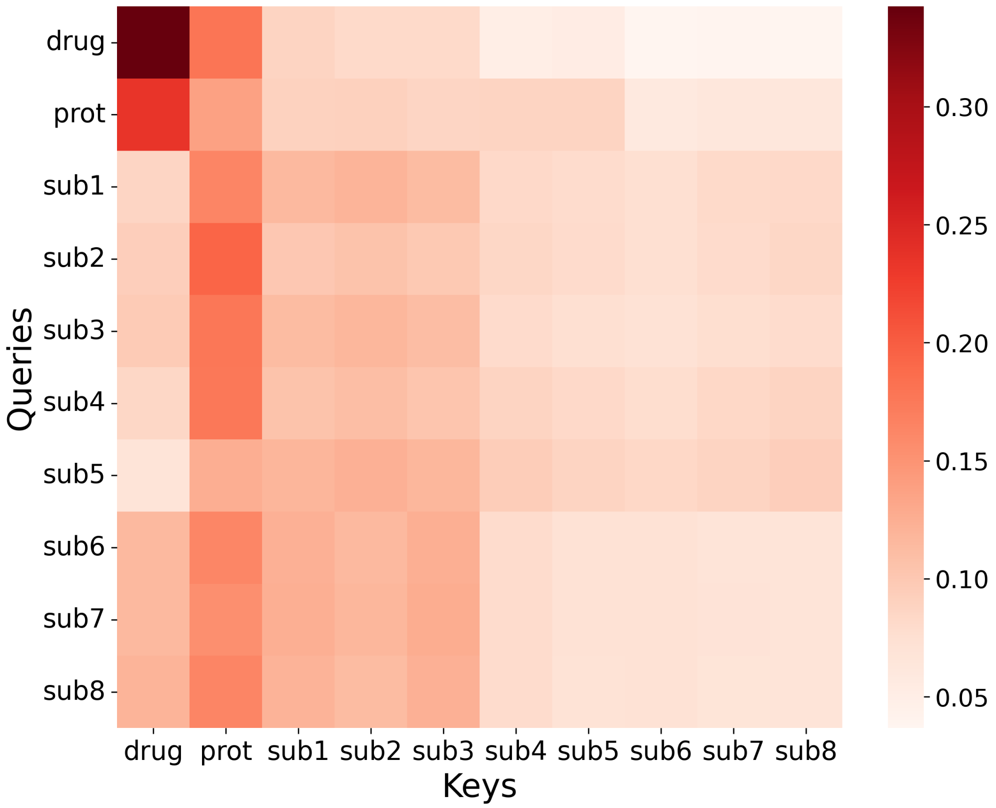

Fig. S2. Attention scores for SP-DTI with HIV protease D545701 as the protein and GW0385 as the ligand. The labels "Drug" and "Protein" represent global embeddings, while "Sub_i" denotes embeddings for subpockets.

REFERENCES

1. T. Voitsitskyi, R. Stratiichuk, I. Koleiev, *et al.*, "3dprotdta: A deep learning model for drug-target affinity prediction based on residue-level protein graphs," RSC Adv. **13**, 10261–10272 (2023).
2. C. Lu, Q. Liu, C. Wang, *et al.*, "Molecular property prediction: A multilevel quantum interactions modeling perspective," Proc. AAAI Conf. on Artif. Intell. **33**, 1052–1060 (2019).
3. K. T. Schütt, H. E. Sauceda, P.-J. Kindermans, *et al.*, "Schnet – a deep learning architecture for molecules and materials," The J. Chem. Phys. **148** (2018).
